# Evaluating the impact of depression, anxiety & autonomic function on health related quality of life, vocational functioning and health care utilisation in acute coronary syndrome patients: the ADVENT study protocol

**DOI:** 10.1186/1471-2261-13-103

**Published:** 2013-11-17

**Authors:** John C Oldroyd, Sheila Cyril, Bhanuja S Wijayatilaka, Adrienne O’Neil, Dean P McKenzie, Silva Zavarsek, Kristy Sanderson, David L Hare, Aaron J Fisher, Andrew B Forbes, C Barr Taylor, David M Clarke, Ian T Meredith, Brian Oldenburg

**Affiliations:** 1 Global Health and Society Unit, Department of Epidemiology and Preventive Medicine, Monash University, Melbourne, VIC 3004, Australia; 2 IMPACT Strategic Research Centre, School of Medicine, Deakin University, Geelong, VIC 3220, Australia; 3 Department of Epidemiology and Preventive Medicine, Monash University, Melbourne, VIC 3004, Australia; 4 Centre for Health Economics, Monash University, Clayton, VIC 3800, Australia; 5 Menzies Research Institute, University of Tasmania, Hobart, TAS 7000, Australia; 6 University of Melbourne School of Medicine, Parkville, Melbourne, VIC Australia; 7 University of California, Berkeley, CA 94720, USA; 8 School of Medicine, Stanford University, Stanford, CA 94305, USA; 9 Department of Psychiatry, Monash University, Monash Health, Clayton, VIC 3168, Australia; 10 MonashHeart, Monash Health, Clayton, VIC 3168, Australia

**Keywords:** Depression, Anxiety, Heart rate variability, Protocol

## Abstract

**Background:**

Depression and anxiety are highly prevalent and co-morbid in acute coronary syndrome patients. Somatic and cognitive subtypes of depression and anxiety in acute coronary syndrome have been shown to be associated with mortality although their association with patient outcomes is unknown, as are the mechanisms that underpin these associations. We are conducting a prospective cohort study which aims to examine in acute coronary syndrome patients: (1) the role of somatic subtypes of depression and anxiety as predictors of health related quality of life outcomes; (2) how somatic subtypes of depression and anxiety relate to long term vocational functioning and healthcare utilisation; and (3) the role of the autonomic nervous system assessed by heart rate variability as a moderator of these associations.

**Methods:**

Patients are being screened after index admission for acute coronary syndrome at a single, high volume centre, MonashHeart, Monash Health, Victoria, Australia. The inclusion criterion is all patients aged > 21 years old and fluent in English admitted to MonashHeart, Monash Health with a diagnosis of acute coronary syndrome. The primary outcome is mean health related quality of life (Short Form-36) Physical and Mental Health Summary scores at 12 and 24 months in subtypes with somatic symptoms of depression and anxiety. Depressive domains are assessed by the Beck Depression Inventory II and the Cardiac Depression Scale. Anxiety is measured using the Speilberger State-Trait Anxiety Inventory and the Crown Crisp Phobic Anxiety questionnaire. Secondary outcomes include clinical variables, healthcare service utilisation and vocational functioning.

**Discussion:**

This manuscript presents the protocol for a prospective cohort study which will investigate the role of somatic subtypes of depression and anxiety as predictors of health related quality of life, long-term vocational functioning and health service use, and the role of the autonomic nervous system in moderating these associations. Findings from the study have the potential to inform more effective pharmacological, psychological and behavioural interventions and better guide health policy on the use of health care resources.

## Background

After ACS, major depression has a prevalence of over 10%, about twice that found in the general population. Less severe symptoms of depression are found in 20-30% of patients after ACS, with similar prevalence rates for anxiety [1-4]. Depression and anxiety have both been shown to result in increased mortality and morbidity [5-9], reduced HRQoL [10], poorer vocational outcomes [11], and delays in returning to work [12]. Despite the high prevalence of depression and anxiety the conditions remain under-recognised, poorly diagnosed and undertreated in ACS populations [13]. Emerging evidence suggests that different combinations of symptoms, representing different subtypes of depression, may result in different outcomes. For instance, there is evidence that depression dominated by somatic symptoms may have worse outcomes than depression dominated by cognitive symptoms. Somatic subtypes of depression, (which include symptoms of fatigue, altered sleeping patterns or changes in appetite) predict cardiovascular prognosis [12] and all-cause mortality [13]. There are indications that somatic subtypes of anxiety disorder may predict coronary heart disease (CHD) [14]. Also, the anhedonia (loss of pleasure or interest) component of depression identifies risk of major adverse cardiac events and all-cause mortality beyond that of major depressive episode and depressive symptom severity in ACS patients [15]. Importantly, key depression subtypes, regardless of whether or not diagnostic thresholds for a disorder are satisfied, have the potential to predict important patient outcomes such as long-term functioning and recovery [16]. Patient outcomes are of particular importance given that HRQoL has been shown to be as important for ACS patients as any gains in survival [13]. Identifying the key symptom subtypes related to these functional outcomes has the potential to guide development and delivery of more targeted and effective interventions.

Not surprisingly, the psychophysiological mechanisms that underpin the relationships between different subtypes of depression and patient outcomes also remain poorly understood. Of particular significance is the role of the autonomic nervous system. Heart rate variability (HRV) reflects the normal healthy autonomically-regulated beat-to-beat variation [17]. In general, healthy persons have more variability in heart rate than unwell persons. Alterations to normal HRV have been shown to be important markers of prognosis among post-myocardial infarction (MI) patients with both depressive and anxiety disorders [18]. Indeed, there is now relatively strong evidence that overall HRV is consistently lower in depressed patients with CHD than in non-depressed patients with CHD [19]. In a large study exploring the association between HRV in patients after MI (n = 805), HRV was significantly lower in the depressed patient group, compared to the non-depressed group, even after adjusting for possible confounders such as diabetes and smoking [20]. Furthermore, there is some evidence to suggest a variation in HRV abnormalities between different depression subtypes in patients with CHD [21]. For example, depression dominated by somatic symptoms, including symptoms of fatigue and psychomotor changes, have been associated with reduced HRV while cognitive symptoms, such as negative self-image, have not [22]. In addition, a smaller group of studies have suggested HRV is lower in CHD patients with anxiety disorders, compared to those without [18]. A recent review also found that HRV has clinically significant prognostic value in depressed patients who have had a recent MI [19]. However, few studies have investigated the relationship between depression dominated by somatic symptoms on patient outcomes such as HRQoL, vocational functioning and health care utilisation. There is also little evidence about whether HRV moderates these associations, that is, alters the strength of the presumed causal relationship between somatic subtypes of depression and patient outcomes. This is a major gap in knowledge given the importance of these outcomes to patients following cardiac events.

In relation to patient outcomes, intervention studies have shown improvements in HRQoL in ACS patients with depression and anxiety. For example, the Managing Co-morbid Depression: Coronary Aftercare Randomised Evaluation (MOOD-CARE) [23] demonstrated improvements in HRQoL following a telephone-counselling program for the management of depression and CHD secondary prevention for ACS patients. After six months follow-up, the intervention resulted in statistically significant improvements in Short Form 12 (SF-12) Mental Component Summary scores (mean difference in change between groups = 5.7; p = 0.041) for those with a history of depression when compared with usual care [24]. Similarly, the Proactive Heart trial [25] used telephone coaching risk factor management interventions in ACS patients following a cardiac event. This trial also demonstrated significant improvements in HRQoL as a result of the intervention in the Mental Component Summary score (p = 0.02), and the Social Functioning (p = 0.04) and Role-Emotional (p = 0.03) subscales of the Short Form 36 (SF-36) compared with usual care. However, both studies used relatively brief measures of depression and anxiety (e.g. Patient Health Questionnaire 9 in MOOD-CARE; Hospital Anxiety and Depression Scale in the Proactive Heart trial), which included few items concerned with feelings of helplessness, worthlessness and hopelessness. Additionally, neither study measured HRV, thus precluding a thorough investigation of depression and anxiety subtypes.

In relation to employment status and health care utilisation, depression and anxiety have been found to be independent predictors of vocational functioning following ACS [26]. For example, those experiencing depression report poorer rates of return to work following ACS [27] as well as poorer productivity [12,28]. Similarly, depression has also been associated with a 15% to 53% increase in 5-year cardiovascular costs in those with myocardial ischaemia [29]. The presence of depressive symptoms exhibited during a hospital stay have been associated with an increased rate of hospital readmissions in medical patients [30]. However, much less is known about healthcare utilisation in ACS patients where symptoms of depression and anxiety occur co-morbidly. A more comprehensive investigation of the predictive relationship of symptom subtypes of depression and anxiety in ACS patients with HRQoL, vocational functioning and health care utilisation has not yet been conducted. This paper presents the study protocol for a prospective cohort study that is in progress. We hypothesise that, ACS patients with the somatic symptom subtype and lower HRV will: 1) report poorer HRQoL (SF-36); 2) report poorer vocational outcomes; and 3) exhibit differing patterns of healthcare service utilization at 12 and 24 months compared to ACS patients without the somatic subtype of depression and with higher levels of HRV at baseline.

## Methods

**Figure 1:**
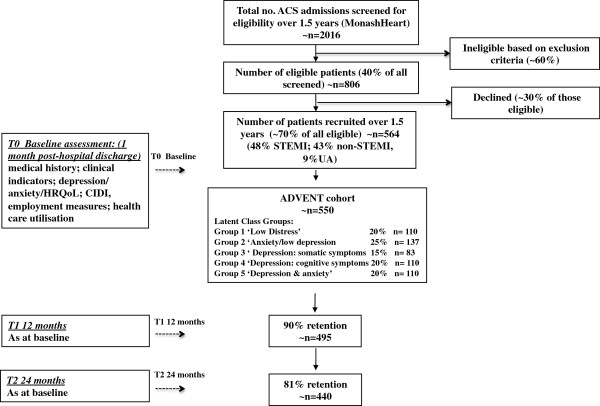
Flow diagram of study.

We are currently enrolling ACS patients into a prospective cohort study in which participants complete an assessment at baseline, 12 and 24 months follow-up (Figure 1).

### Study aims

In ACS patients, we aim to examine: (1) the role of somatic subtypes of depression and anxiety as predictors of HRQoL outcomes; (2) how somatic subtypes of depression and anxiety relate to long term vocational functioning and healthcare utilisation; and (3) the role of the autonomic nervous system assessed by HRV as a moderator of these associations.

### Study sample

#### Eligibility criteria

Eligibility criteria includes: (i) aged > 21 years old; (ii) admitted to MonashHeart, Monash Health with a diagnosis of ACS (ST-elevation MI, non-ST-elevation MI or unstable angina) within 4 weeks; (iii) an adequate understanding of English sufficient to complete the study. Patients are excluded if they are: unable to give informed consent, pregnant, cognitively impaired, report substance abuse, have a terminal illness and/or other illness that may impair participation, participating in other trials.

### Sample recruitment procedures

We are recruiting 550 ACS patients over 18 months from the MonashHeart, Monash Health in the south-eastern suburbs of Melbourne, Victoria (catchment population ~1.2 million). This provides the largest network of cardiac health services in Australia (>1340 ACS admissions/year). Participants are being recruited between January 2013 and June 2014. Recruitment is performed by study co-ordinators employed by MonashHeart, Monash Health. Study co-ordinators screen admitted patients for eligibility by approaching those deemed suitable, explaining the study and obtaining informed consent. A letter is mailed to participant’s primary care provider/s informing them of the study aims and the participant’s agreement to enrol in the study. Shortly after enrolment, participants are sent a ‘welcome pack’ containing a welcome letter and further details about the study. To facilitate a comparison of participants and non-participants, de-identified demographic data (year of birth, sex, cardiac diagnosis on admission) are being collected on the entire population of eligible ACS patients identified during the study period.

### Primary endpoint and sample size calculation

Power and sample size calculations are based on detecting differences in mean SF-36 Physical and Mental Health Summary scores at 12 and 24 month follow-up assessment between the symptom subgroups as determined by latent classes at baseline (see ‘Subtype classification - latent classes’ paragraph and ‘Data analysis’ description below). A clinically significant change in standardised (mean = 50, standard deviation (SD) = 10) summary scores on the SF-36 is often regarded as 5 units [31] or an effect size of 0.50, corresponding to what is by convention labelled a moderate effect size [32]. Depression is generally associated with an even larger effect size. For example, a study of MI patients [33] found a 10 unit (effect size = 1.0) difference in 12 month SF-36 scores between those with low and high scores on a measure of depression at baseline. Therefore, an expected effect size of 0.60 between the groups with the lowest expected mean score (group 1), and that of each of the three groups with the next lowest mean score (groups 2, 3, 4), would appear to be reasonable, together with an effect size of 1.0 between the lowest and highest groups (1 and 5). With 5 latent classes there are 10 pairwise comparisons, therefore all comparisons are performed using a 0.5% significance level to ensure an overall Type I (false positive) error rate of 5% (Bonferroni correction). The within-group SD is assumed to be 10 SF-36 units, as above. Since group 3 is expected to be the smallest group size (15% of sample), sample size is determined to have the comparison between groups 1 and 3 being able to detect a difference of 6 SF-36 units with 80% power. This requires 88 patients in group 1 and 66 in group 3. From these numbers, the sizes of the other groups are determined, resulting in a total of 440 completing patients being required. An expected attrition rate of ~10% per year indicates that the number of patients required to be recruited is ~550 (expected group sizes would be 110, 137, 83, 110, 110 for groups 1–5, respectively) (Figure 1). These group sizes ensure the power to detect a difference of at least 5 units is 92% for a comparison between groups 1 and 2, 80% between groups 1 and 3, and 88% between groups 1 and 4. An effect size of 10 SF-36 units between groups 1 and 5 can be detected with over 99.9% power. In terms of precision of group comparisons, any pairwise comparison will have a Bonferroni 95% confidence interval width of at most +/− 3.5 SF-36 units. The detection of moderation of the relationship between the 5 latent classes and SF-36 by HRV will involve assessing the additional explained variation resulting from adding the appropriate statistical interaction terms into a regression-model containing the main effects of the latent classes and HRV. Assuming conservatively that 10% of variation in SF-36 is explained via these main effects and other prognostic covariates, the sample size of 440 completing subjects has 80% power to detect an increase in explained variation of 2.4% from the addition of the (4 degrees of freedom) interaction terms. This corresponds to an effect size of 0.027, which is conventionally regarded as small [32]. For detection of variation across latent classes in the employment status at 12 months or for a binary health services utilisation measure, expected absolute differences of the order of 20-25% between any pair of latent classes (with Bonferroni correction) can be detected with 80% power.

### Ethics approval

The study has been approved by the Human Research Ethics Committee at Monash Health (HREC Ref 12249B) and Monash University (Project Number CF12/3610 – 2012001723).

### Data collection and outcome measures

Baseline data are collected during admission for ACS by the study co-ordinators (demographic data) and Research Assistants (clinical data measured during face-to-face clinic assessments as well as questionnaire data collected by computer assisted telephone interview (CATI)). Follow-up data (as at baseline) are being collected by Research Assistants after 12 and 24 months (Table 1).

**Table 1:** Measurements at baseline and during follow-up

**Outcome variable**	**Measurement tool/Data collection method**	**Time-point (months)**		
		**0**	**12**	**24**
***Primary outcome***				
Difference in mean HRQoL (SF-36) Physical and Mental Health Summary scores at 12 and 24 month follow-up between the latent classes of depression and anxiety determined at baseline	Short Form 36 [47]	✓	✓	✓
		✓	✓	✓
***Secondary outcomes***				
*Medical history*				
Co-morbidities	Medical records	✓		
Medications	Self-report discharge scripts	✓	✓	✓
*Clinical indicators*				
Serum lipids; HbA1c; FBG; hs-CRP	Medical records	✓		
Blood pressure; pulse	Clinical assessment	✓	✓	✓
Waist circumference	Clinical assessment	✓	✓	✓
Body Mass Index	Clinical assessment	✓	✓	✓
Diabetes	Self-report diagnosis; HbA1c	✓	✓	✓
Heart Rate Variability	Clinical assessment	✓	✓	✓
*Depression/anxiety*				
Current depressive symptoms	BDI-II [40] & CDS [42]	✓	✓	✓
Current anxiety symptoms	STAI-S & STAI-T [43]	✓	✓	✓
Phobic Anxiety	Crown Crisp Index[45]	✓	✓	✓
Depression/anxiety history	CIDI- Auto 2.1 [50]	✓	✓	✓
*Behavioural measures*				
Worry	The Penn State Worry Short Form [48]	✓	✓	✓
Sleep	Insomnia Severity Index [49]	✓	✓	✓
Work related outcomes	Self-report	✓	✓	✓
*Economic measures*				
Preference-based measure of HRQoL (SF-6D)	Short Form 36 [47]	✓	✓	✓
Health care utilisation	Self-report	✓	✓	✓

### Baseline

The following *demographic data* are collected during admission: age, sex, medical history including diagnosis on admission and co-morbidities (including hypertension, stroke, cancer, diabetes and mental illness). The presence of atrial fibrillation, flutter, arrhythmia or insertion of a permanent pacemaker is noted. *Blood measurements* (fasting blood glucose, lipids (cholesterol, triglycerides, high-density lipoprotein cholesterol (HDL-C), low density lipoprotein cholesterol (LDL-C)), high sensitivity C-reactive protein (hs-CRP), and glycosylated haemoglobin (HbA1C)) during admission are recorded.

Clinical measurements are collected during attendance at a clinical assessment. These include *anthropometry* (weight, height, body mass index, waist circumference), *blood pressure*, pulse and *heart rate variability*. Medication history is collected from hospital discharge scripts or from self-report of medications (verified by Research Assistants using packets/boxes of medications brought in by participants). CATIs are performed to collect questionnaire data including socio-demographic data (country of birth, language spoke at home, medical insurance cover, annual income band, highest education qualification, employment at time of cardiac event); BDI-II, CDS, STAI-Y*,* Crown Crisp Phobic Anxiety questionnaire, SF-36, Penn State Worry Questionnaire Short Form, Insomnia Severity Index and Composite International Diagnostic Interview (CIDI). Data on employment functioning post-ACS and health care utilisation in the previous 6 months is also collected.

### Follow-up

Clinical measurements are collected during a face to face clinical assessment at 12 and 24 months following discharge from hospital. These are the same as those at baseline. In addition, CATI’s are performed to collect questionnaire data at 12 and 24 months which are the same as at baseline except health care utilisation in which data from the previous 12 months will be recorded.

### Details of the measurements

#### Blood measurements

Blood measurements include fasting blood glucose, lipids, hs-CRP and HbA1C. Blood samples are assayed by Monash Health Pathology. hs-CRP (mg/L) is assayed by Roche/Hitachi Modular® System P. HDL-C is being measured using a commercial enzymatic assay using a Beckman Coulter DXC800 analyzer with reagents supplied by Beckman Coulter Diagnostics **(**Sydney, Australia). Triglycerides are being measured by standard methods within Southern Health Pathology. LDL-C is being calculated using the Friedewald Formula [34]. HbA1C is assayed using high performance liquid chromatography (ADAMS ARKRAY Glycohaemoglobin analyzer HA8160, Integrated Sciences, Australia CV% 1.5- 1.7).

#### Anthropometry

Weight is measured to the nearest 0.1 kg on standing digital scales (SECA 813 Digital Scales) placed on a level, hard surface without shoes and in light clothing. Height is measured using a wall mounted stadiometer. Body mass index is calculated and World Health Organization cutoffs applied namely, overweight **≥**25 kg/m^2^; obese ≥30 kg/m^2^[35]. Waist circumference is measured using a spring loaded tape measure (SECA 201) at the mid-point between the lower costal margin and the iliac crest and World Health Organization cutoffs applied (increased risk men 94 cm; women 80 cm) [35,36].

#### Blood pressure

Blood pressure (BP) is measured using an automated device (OMRON HEM 7221 Automatic BP Monitor) calibrated as per manufacturer’s instructions. The average of two readings of systolic and diastolic BP is used. Hypertension is defined using World Health Organization cut off points namely, systolic BP ≥140 mmHg or diastolic BP ≥90 mmHg [37]. The average of two readings of pulse is recorded from the BP monitor.

#### Heart rate variability

HRV [38] is collected via an electrocardiograph (ECG) recording [39]. A dedicated computer is used with AD Instruments software (LabChart v7.3 for Windows) for continuous beat-to-beat heart rate recording with detection of R waves at frequency of 1000 Hz. Ectopic beats, artefacts, patients with atrial fibrillation, flutter, permanent pacemakers in situ or with less than 95% acceptable R-R intervals are excluded from the HRV analyses.

Heart rate variability recording & analysis:

● Participants are asked to avoid caffeine, heavy physical activity, smoking and alcohol 10 hours preceding the clinic visit. Participants are asked to have breakfast or at least a snack approximately 1 hour before the clinic visit (protocol violations are recorded however participants are not excluded if these conditions are not met).

● Study procedures are explained to patients in advance and they are asked to lie quietly and still before and during the recording. All attempts are made to ensure that testing is conducted in a consistent environment as stressors such as arousal level, temperature and circadian rhythm can alter HRV measures. Temperature is standardised and time of recording is documented. Patients are connected to a three lead electrocardiogram (ECG) in the supine position then rested for 10 minutes. A 20-minute ECG recording is then obtained.

● Data are converted to R-R intervals for time domain measurement and power spectrum (frequency) analysis. Time domain measures include mean R-R interval duration (RR (ms)) (in which the ‘R-R interval duration’ is defined as the time period between successive R waves), standard deviation of all normal to normal R-R intervals (SDNN (ms)), the root mean square of successive differences between normal-to-normal R-R intervals (rMSSD (ms)). Frequency domain measures are generated through fast Fourier transformation to produce a power spectral density curve (a plot of the frequency of the oscillations of variation in R-R interval duration against the square of their amplitude): high frequency (ms^2^) is area under the power spectral density curve from 0.15-0.50 Hz (vagal modulation) low frequency (ms^2^) is area under the power spectral density curve from 0.04-0.15 Hz (parasympathetic and sympathetic modulation). LF/HF ratio is also calculated as a stronger measure of sympathovagal balance. Adjustments are made using artefact identification software to flag potential artefact that are manually reviewed (using Poincaré plots) and deleted if appropriate. All files are analysed in blinded fashion.

#### Beck depression interview

The BDI-II is a twenty one item multiple choice self-report inventory that provides an overall measure of somatic and cognitive symptoms of depression. It is designed for individuals aged 13 years and over and has been used in cardiac populations [40]. It is composed of items relating to symptoms of depression such as hopelessness and irritability, cognitions such as guilt or feelings of being punished, as well as physical symptoms such as fatigue, weight loss and lack of interest in sex.

#### Cardiac depression scale

The CDS is a twenty six item questionnaire for assessing depression in cardiac patients (alpha reliability coefficient 0.90) [41]. It correlates well with clinical rating and with the BDI-II. Cognitive subscales will be used to measure anhedonia (loss of interest or pleasure) and hopelessness [42].

#### Speilberger state-trait anxiety inventory

The STAI-Y is a widely used instrument to measure transient and enduring levels of anxiety [43]. It has been used to distinguish between anxiety and depression symptoms and chronic or trait anxiety subtypes in cardiac populations [44]. The instrument is divided into two sections (STAI-S & STAI-T) each with twenty questions. It contains four point Likert response items per question.

#### Crown-crisp index of phobic anxiety

The Crown-Crisp Index of Phobic Anxiety is an eight item questionnaire used to measure phobic anxiety [45]. Phobic anxiety has been strongly associated with risk of fatal CHD [46].

#### Health related quality of life

The SF-36 (Version 2) is a multipurpose, multidimensional health status scale for measuring HRQoL. It comprises eight scales measuring Physical Function, Role Physical, Bodily Pain, General Health, Vitality, Social Function, Role Emotional, and Mental Health [47]. These scales are collapsed into two summary scales, the Physical Component Scale (PCS) and the Mental Health Component scale (MCS).

#### Penn state worry questionnaire short form

The Penn State Worry Questionnaire Short Form is a seven item instrument to measure worry [48]. It possesses high internal consistency and good test-retest reliability.

#### Insomnia severity index

The Insomnia Severity Index is a seven item questionnaire to assess insomnia severity [49]. The psychometric properties show it has adequate internal consistency and is a reliable self-report measure to evaluate perceived sleep difficulties. It is also a valid and sensitive measure to detect changes in perceived sleep difficulties with treatment.

#### Composite international diagnostic interview (CIDI)

The CIDI Auto version 2.1 is a computer-assisted, comprehensive, fully-standardized interview that can be used to assess mental disorders and provide diagnoses according to the definitions and criteria of the tenth revision of the *International Classification of Diseases* (ICD-10) (World Health Organization 1992, 1993) and the fourth edition of the American Psychiatric Association’s *Diagnostic and Statistical Manual of Mental Disorders* (DSM-IV) (American Psychiatric Association 1994) [50]. It is designed to be used by trained lay interviewers and is used in this study to assess history of clinical diagnosis of depressive and anxiety disorders.

#### Economic evaluation

Employment, medication and health services utilisation are collected through both self-report data and linkage with Medicare Australia, and Pharmaceutical Benefits Scheme data. This allows us to identify the relationship between particular depression and anxiety subtypes and healthcare use and labour force outcomes. We are also examining the relationship between HRQoL and labour force outcomes. Additionally, the data are matched to other population data to compare HRQoL differences. Furthermore, we are using these data to calculate the societal impact of these conditions/symptoms by linking health care use, to labour force status and to HRQoL.

### Subtype classification - latent classes

A recently developed statistical technique, *latent class analysis* (LCA) [51], is used to determine depression/anxiety *subtypes*. This has recently been employed with somatic and cognitive symptoms of depression and anxiety in a variety of populations [51,52], including the medically ill [16]. Unlike variable-centred approaches such as factor analysis, LCA is a person-centred approach; it allows grouping of individuals into *classes* (subtypes), on the basis of shared characteristics such as depression and anxiety symptoms. It can therefore be readily ascertained which particular individuals have certain combinations of symptoms. LCA allows probabilistic assignment of each individual to each class although in practice, individuals are often assigned to the class with the highest probability [53]. Classes can be compared on outcomes such as HRQoL and vocational functioning, while controlling for risk factors such as pre-ACS depression and anxiety, blood pressure, as well as CIDI diagnoses of depression and anxiety. Based upon prior latent class analyses of similar populations [16,54], we expect to find five classes: 1) ‘low distress’ (low on all or most dimensions), consisting of 20% of patients, 2) ‘anxiety but low depression’ (25%), 3) ‘predominantly depression with somatic symptoms’ (15%), 4) ‘predominantly depression with cognitive symptoms’ (20%), 5) ‘comorbid depression and anxiety’ (high on most or all dimensions) (20% of patients).

### Primary outcome

The primary outcome is difference in mean HRQoL (SF-36) Physical and Mental Health Summary scores at 12 and 24 months between latent class group 1 (‘low distress’ i.e. low somatic symptoms of depression and anxiety assessed by BDI-II, CDS, Crown Crisp and STAI-Y and high/normal HRV, consisting of 20% of participants) and latent class group 3 (‘predominantly depression with somatic symptoms’ and low HRV, 15% of participants), determined at baseline. Secondary outcomes include clinical variables, healthcare service utilisation and vocational functioning.

### Data analysis

Hypotheses 1–3 are utilising LCA of symptom subtypes of depression and anxiety, as measured using the BDI-II, CDS, Crown Crisp and STAI-Y [51]. The resulting classes of patients are then compared at baseline, 12 and 24 months on outcome measures including HRQoL, health service utilisation and occupational functioning, using appropriate regression techniques [55]. Moderation of the effects of the LCA classes on outcomes by HRV will be assessed with the inclusion of appropriate interaction terms between LCA classes and HRV in the regression models. Patterns of change in outcome over time are assessed using generalized estimating equations to account for the repeated measurements of individuals over time. The employment and health resource outcomes specified in hypotheses 2 and 3 are being modelled using econometric techniques [56], to analyse the relationship of latent classes defined by profiles of depression/anxiety symptoms, with healthcare use and vocational functioning. A separate analysis linking this to patients’ healthcare service use is being explored using systems of equations, where the relationship between variables is accounted for simultaneously, so the effects of latent class (subtype) membership on HRQoL, employment status, and health care utilisation can be estimated at the same time, across time periods.

## Discussion

Depression and anxiety in ACS patients pose a significant burden to patients and health care services. Studies investigating the association of cognitive and somatic symptoms of depression and anxiety with long-term quality of life and vocational outcomes in ACS patients are lacking. Further, there is little data on the psychophysiological mechanisms underpinning these associations. This manuscript presents the protocol for a prospective cohort study that will investigate the role of somatic subtypes of depression and anxiety as predictors of HRQoL, long-term vocational functioning and health service use, and the role of the autonomic nervous system in moderating these associations. A greater understanding of these associations is needed to develop more effective pharmacological, psychological and behavioural interventions and to better guide health policy on the use of health care resources. The study is aligned with Australian National Health Priorities seeking to address mental illness and chronic diseases.

## Abbreviations

HRQoL: Health related quality of life; ACS: Acute Coronary Syndrome; HRV: Heart rate variability.

## Competing interests

The authors declare that they have no competing interests.

## Authors’ contributions

JO is undertaking data collection and led in writing the paper. SC and BW are undertaking data collection and revised drafts of the paper. AON, DMcK, SZ, KS, DH, CBT, DC, IM, BO designed the study and co-wrote the paper. A Forbes designed the statistical analysis. A Fisher undertook sample size calculations. All authors contributed to, read and approved the final manuscript.

## Pre-publication history

The pre-publication history for this paper can be accessed here:

http://www.biomedcentral.com/1471-2261/13/103/prepub
